# *In Vitro* Exposure to Prostratin but Not Bryostatin-1 Improves Natural Killer Cell Functions Including Killing of CD4^+^ T Cells Harboring Reactivated Human Immunodeficiency Virus

**DOI:** 10.3389/fimmu.2018.01514

**Published:** 2018-06-29

**Authors:** Maria Giovanna Desimio, Erica Giuliani, Angelo Salvatore Ferraro, Gaspare Adorno, Margherita Doria

**Affiliations:** ^1^Laboratory of Immunoinfectivology, Immune and Infectious Diseases Division, Bambino Gesù Children’s Hospital, IRCCS, Rome, Italy; ^2^SIMT, Policlinico Tor Vergata, Rome, Italy; ^3^Department of Biomedicine and Prevention, Università degli Studi di Roma Tor Vergata, Rome, Italy

**Keywords:** HIV, latency-reversing agents, protein kinase C agonists, prostratin, bryostatin-1, natural killer cell function, antibody-dependent cellular cytotoxicity, NKG2D

## Abstract

In the attempt of purging the HIV-1 reservoir through the “shock-and-kill” strategy, it is important to select latency-reversing agents (LRAs) devoid of deleterious effects on the antiviral function of immune effector cells. Here, we investigated two LRAs with PKC agonist activity, prostratin (PRO) and bryostatin-1 (BRY), for their impact on the function of natural killer (NK) cells, the major effectors of innate immunity whose potential in HIV-1 eradication has emerged in recent clinical trials. Using NK cells of healthy donors, we found that exposure to either PRO or BRY potently activated NK cells, resulting in upmodulation of NKG2D and NKp44 activating receptors and matrix metalloprotease-mediated shedding of CD16 receptor. Despite PRO and BRY affected NK cell phenotype in the same manner, their impact on NK cell function was diverse and showed considerable donor-to-donor variation. Altogether, in most tested donors, the natural cytotoxicity and antibody-dependent cellular cytotoxicity (ADCC) of NK cells were either improved or maintained by PRO, while both activities were impaired by BRY. Moreover, we analyzed the effect of these drugs on the capacity of treated NK cells to kill autologous latently infected CD4^+^ T cells reactivated *via* the same treatment. First, we found that PRO but not BRY increased upmodulation of the ULBP2 ligand for NKG2D on reactivated p24^+^ cells. Importantly, we showed that clearance of reactivated p24^+^ cells by NK cells was enhanced when both targets and effectors were exposed to PRO but not to BRY. Overall, PRO had a superior potential compared with BRY as to the impact on key NK cell functions and on NK-cell-mediated clearance of the HIV-1 reservoir. Our results emphasize the importance of considering the effects on NK cells of candidate “shock-and-kill” interventions. With respect to combinative approaches, the impact on NK cells of each LRA should be re-evaluated upon combination with a second LRA, which may have analogous or opposite effects, or with immunotherapy targeting NK cells. In addition, avoiding co-administration of LRAs that negatively impact ADCC activity by NK cells might be essential for successful application of antibodies or vaccination to “shock-and-kill” strategies.

## Introduction

In HIV-1-infected individuals, antiretroviral therapy (ART) drastically reduces plasma viral loads and hinders clinical progression, yet it fails to totally eradicate the virus that persists in a transcriptionally quiescent form in latently infected cellular reservoirs such as resting memory CD4^+^ T cells (1, 2). From HIV-1 reservoirs, the virus rapidly rebounds once ART is interrupted (3); hence, patients must stay on therapy for life and face several long-term health risks. For these reasons, major efforts have been made in recent years to identify strategies for eradicating HIV-1 reservoirs through the activation of provirus transcription followed by elimination of cells harboring reactivated virus by viral cytopathic effects or by the host immune system, an approach referred to as “shock-and-kill” (4). Various drugs belonging to distinct functional categories, such as histone deacetylase inhibitors (HDACis) and protein kinase C agonists (PKCas), serve *in vitro* as HIV-1 latency-reversing agents (LRAs) in T cell lines and primary CD4^+^ T cell models. In fact, by acting at the level of chromatin organization or *via* the PKC signaling pathway, respectively, HDACi and PKCa elicit the recruitment of activating transcription factors (e.g., NF-κB, AP-1, and NFAT) at the HIV-1 long terminal repeat (LTR) region, leading to virus reactivation [reviewed in Ref. (5, 6)]. In addition, HDACi and PKCa can stimulate HIV-1 transcription through increased expression and/or recruitment at the viral promoter of positive transcription elongation factor b (P-TEFb) (7, 8). Of note, among several tested LRAs, only PKCas are effective at inducing *ex vivo* HIV-1 transcription in cells isolated from ART-treated aviremic patients (9–11).

Unfortunately, initial clinical trials in which HDACis (i.e., Vorinostat—SAHA—Panobinostat, and Romidepsin) were administered to patients on ART found no, or only modest, reduction of the HIV-1 reservoir size despite increased levels of cell-associated HIV-1 RNA (12–14). Alongside, various studies provided evidence that cytotoxic CD8^+^ T cell (CTL) responses of patients cannot efficiently clear infected cells after the reversal of latency, likely due to the low frequency or poor functionality of HIV-1-specific CTLs (15, 16) and/or to the accumulation of CTL escape mutations within latent HIV-1 genomes (17). Moreover, HDACis were shown to suppress *in vitro* the function of CTLs, hence inhibiting their capacity to eliminate HIV-infected CD4^+^ T cells (18–20). At present, bryostatin-1 (BRY), a natural macrocyclic lactone clinically used as an anticancer agent (21), is the only PKCa that has been administered to ART-treated patients (22). However, in this pilot trial implying a single dose of BRY, neither PKC activation nor transcription of latent HIV-1 were induced, thus new trials assessing higher doses and/or multiple administrations of BRY are needed. Other notable PKCas that, analogously to BRY, are effective at reactivating latent HIV-1 *ex vivo* but have not yet been tested for this activity *in vivo*, include prostratin (PRO), a non-tumor promoting phorbol ester isolated from plants, and derivatives of ingenol ester (10, 11).

Overall, results from pilot HIV-1 eradication studies suggest that boosting the antiviral immune response of patients might be essential to eliminate the viral reservoirs reactivated by LRAs. Indeed, various immunotherapies that may be combined with LRA administration are been developed, including HIV-1 vaccines, monoclonal antibodies (mAbs) engaging Fcγ-receptor bearing cells to mediate antibody-dependent cellular cytotoxicity (ADCC), and dual-affinity retargeting proteins that direct CTL killing of infected target cells [reviewed in Ref. (23, 24)]. In addition, recently reported evidences suggest that natural killer (NK) cells, the major effector cells of the innate immune system, have an important role, possibly superior to that of CTLs, in the context of HIV-1 eradication strategies. A key feature of NK cells is their constitutive cytotoxic activity against virus-infected as well as tumor cells that is independent of prior antigen exposure, hence not affected by escape mutants, and is regulated through the balance of signals delivered by activating and inhibitory receptors (25). NK cells can also kill antibody-coated targets *via* ADCC and regulate immune responses *via* cytokines and chemokines production as well as by cell-to-cell interactions (26). Work from various laboratories including our own has shown that HIV-1-infected T cells are exposed to NK cell recognition and killing due to virus-induced upregulation of ligands for the activating NKG2D receptor (27–31), a phenomenon that is maintained also in latently infected CD4^+^ T cells once the virus is reactivated, as we showed in a recent report (32). Of note, in a clinical trial employing Panobinostat to reverse HIV-1 latency in patients on ART, the expansion of activated NK cells, not HIV-1-specific CTLs, was the major correlate of viral DNA decline (33). Moreover, reported results from ongoing clinical trials indicate that latency-reversing treatment with Vorinostat or with a toll-like receptor 9 agonist potently boosts the function of NK cells in ART patients (34–36). Although not yet supported by data from clinical trials, administration of LRAs with PKCa activity may stimulate the function of NK cells. Indeed, extensive experimental evidence based on the use of PKC inhibitors or knockout mice demonstrated that PKC has an essential role in virtually all NK cell functions, including cytotoxicity, ADCC, and IFN-γ production (37–39). One recent report showed that pre-exposure of purified NK cells to PRO did not affect their capacity to kill an established NK-cell tumor target (K562 cells) but increased their capacity to suppress HIV-1 replication *in vitro* in a T cell culture, while treatment with a different PKCa, Ingenol-B, had an inhibitory effect on both functions (40). Although BRY has been used in several trials to treat cancer (21) and its capacity to sensitize acute myeloid leukemia cells to NK-cell-mediated killing has been reported (41), it is not known at present if BRY may directly influence NK cell activity against tumor or infected cells.

As it is critical to understand the impact of PKCas on the function of NK cells needed to clear HIV-1-infected targets, we performed a comprehensive analysis of the effects of PRO and BRY on NK cells purified from healthy subjects. We found that both drugs activate NK cells modifying their phenotype. We also measured the cytotoxicity against tumor targets and ADCC showing that, despite donor-to-donor variations exist, NK cell function is generally maintained, if not enhanced, after exposure to PRO but impaired by BRY. Most importantly, we report that PRO but not BRY enhances NK-cell killing of autologous CD4^+^ T cells harboring reactivated HIV-1 when both effectors and targets are exposed to the drugs *in vitro*.

## Materials and Methods

### Cells, Antibodies, and Reagents

All cells (primary NK and CD4^+^ T cells, K562, Raji) were maintained in complete RPMI 1640 medium supplemented with 10% fetal bovine serum, 0.2 mM l-glutamine, and 100 U/ml penicillin–streptomycin (all from Euroclone).

PBMCs were obtained by Ficoll separation of buffy coats or intravenous blood samples from a donor bank. Ethical committee approval and written informed consent from all participants were obtained, in accordance with the Declaration of Helsinki.

Primary NK and CD4^+^ T cells were isolated from PBMCs by negative selection with Dynabeads Untouched Human NK Cells Kit (Invitrogen–Life Technologies) and EasySep CD4^+^ T-cell Enrichment Kit (Stem Cell Technologies), respectively, according to the manufacturer’s protocol.

The purity of isolated NK (CD3^−^CD56^+^CD16^−/+^) and CD4^+^ T cells (CD3^+^CD4^+^) was assessed by immunolabeling and FACS analysis.

For flow cytometric analysis, the following mouse mAbs were used: CD3/AlexaFluor700 (UCHT1), CD56/PerCpCy5.5 (B159), CD16/BV510 (3G8), CD4/PerCp (L200), CD69/PE (FN50), and conjugated mouse IgG for isotype control staining from BD Pharmingen; CD56/PerCp (MEM-188) from Thermo Fisher Scientific; NKG2D (CD314)/PE (1D11), CD3/APC (UCHT1), and CD16/APC-eFluor780 (CB16) from eBioscience; CD107a/FITC (H4A3), DNAM-1 (CD226)/FITC (11A8), NKp30/PE (P30-15), NKp44/PE (P44-8), and anti-NKp46/PE-Cy7 (9E2) from BioLegend; p24/FITC (KC57) from Beckman Coulter; MICA/B (MAB13001) and ULBP2/5/6 (MAB1298) from R&D Systems. As a secondary antibody, Alexa647-coniugated goat anti-mouse IgG (GAM) (Invitrogen) was used.

The mAbs employed in Western blotting were anti-ADAM17 (111633; R&D Systems) and anti-GAPDH (glyceraldehyde-3-phosphate dehydrogenase) (MAB374; Millipore). As secondary antibody, we used horseradish peroxidase-conjugated GAM (Cell Signaling).

The anti-NKG2D (149810; R&D Systems) mAb and isotype control IgG_1_ were used in cytotoxicity assays.

Where indicated, cells were treated with 10 µg/ml phytohemagglutinin (PHA), 1 or 10 µM PRO (both from Sigma-Aldrich), 5 and 10 nM BRY (Santa Cruz Biotechnology), 12.5 ng/ml IL-15 (Peprotech), 25 µM matrix metalloproteinase inhibitor III (MMPI-III; Calbiochem), and 29 nM CCL19 (R&D Systems).

### Flow Cytometry

To assess viability, cells were stained with the LIVE/DEAD fixable NEAR-IR dead cell stain kit according to the manufacturer’s protocol (Life Technologies). To stain CD107a, cell cultures were supplemented with CD107a/FITC mAb (or control IgG_1_/FITC) and, after the first hour, with Monensin (Golgi stop, diluted 1:1,500; BD Pharmingen) and 10 µg/ml Brefeldin A (Sigma-Aldrich). The following procedures were performed in PBS containing 0.5% BSA and 0.1% NaN_3_. To label cell-surface molecules, cells were incubated for 20 min at 4°C with specific mAbs (if not conjugated, a second incubation with GAM-Alexa647 was performed after a wash). For detection of intracellular p24, cells were fixed and permeabilized with BD Biosciences reagents, then incubated at room temperature for 30 min with p24/FITC mAb. All immunolabeled cells were finally washed, resuspended in 1% paraformaldehyde (PFA), and acquired on a FACSCanto II (BD Biosciences) or Cytoflex (Beckman Coulter). Positive cell gating was set using fluorescence minus one control. Mean fluorescence intensity (MFI) was subtracted of the value obtained with isotype control antibody. Data analyses were performed using Flow Jo software (Tree Star).

### Enzyme-Linked Immunosorbent Assay (ELISA)

Natural killer cells were seeded at 3.5 × 10^6^/ml in complete medium alone or supplemented with 1 µM PRO or 10 nM BRY and cultivated for 5 h. Cell culture supernatant was collected and analyzed with the Human FCGR3A/CD16A ELISA kit (LifeSpan BioScience) following the manufacturer’s instructions to measure sCD16 amounts.

### Western Blotting

Natural killer cells were lysed as described previously (42), then 20 µg of total cell lysate was separated on 10% SDS-PAGE and immunoblotted with primary (specific for ADAM17 and GAPDH) and secondary antibodies. The protein-specific signals were detected with Pierce ECL substrate (Thermo Fisher Scientific) and quantified by densitometry.

### NK-Cell Cytotoxicity Assays

Flow cytometry-based cytotoxicity assays were performed using K562 cells as targets and primary NK cells treated with 10 µM PRO or 5 nM BRY or not treated (nt) for 18 h following a previously described method (43) with minor modifications. Briefly, NK cells were labeled with 5 µM 5,6-carboxyfluorescein diacetate succinimidyl ester (CFSE; Sigma-Aldrich) for 7 min at 37°C, washed twice, then seeded with 2 × 10^5^ K562 cells at different effector-to-target cell (E:T) ratios in a 96-well plate for 4 h at 37°C 5% CO_2_. Then, cells were labeled with 5 µg/ml 7-aminoactinomycin D (7-AAD; Sigma-Aldrich) for 20 min at +4°C, washed, and fixed with 1% PFA. For each E:T ratio, 20,000 target cells (gated as CFSE^−^) were acquired by FACS. The percentage of specific lysis was calculated as follows: 100 × (% 7-AAD^+^ target cells in sample − basal % 7-AAD^+^ target cells)/(100 − basal % 7-AAD^+^ target cells). The specific lysis derived from three E:T ratios per sample was conversed to lytic units (LU), defined as the number of effector cells required to lyse 20% of 2 × 10^5^ target cells (44), and results were expressed as the number of LU contained in 10^7^ NK cells.

### ADCC Assay

PBMCs (effectors) freshly isolated from intravenous blood sample were cultivated for 18 h in medium alone or supplemented with 1 µM PRO or 10 nM BRY, labeled with CFSE as described above, then added to plates (3 × 10^5^/well).

To prepare targets, 5 × 10^5^ Raji cells were pre-coated or not with Rituximab (Rtx; anti-hCD20-hIgG1; InvivoGen) at 20 µg/ml for 30 min on ice. Next, Rtx^+^ or Rtx^−^ Raji were added to CFSE^+^ PBMCs at a 10:1 E:T ratio (3 × 10^4^/well) for 4 h at 37°C 5% CO_2_. Finally, as described above, after 7-AAD labeling, 20,000 CSFE^−^ target cells were acquired by FACS, and the overall percentage of lysis was calculated. The percentage of ADCC was calculated using the following formula: 100 × (% 7-AAD^+^ targets Rtx^+^ with effectors − % 7-AAD^+^ targets Rtx^−^ with effectors)/(% 7-AAD^+^ targets Rtx^+^ with effectors).

### Establishment and Reactivation of Latently Infected CD4^+^ T Cells

Primary CD4^+^ T cells cultures infected with HIV-1 were established and then reactivated as previously described (45) with minor modifications. Briefly, purified CD4^+^ T cells were cultivated with 29 nM CCL19 for 1–3 days. After this period, the quiescent state of the cells was verified by excluding the presence of CD69^+^, CD25^+^, and HLA-DR^+^ cells by FACS analysis. Then, cells were infected by spinoculation with 300 ng p24/10^6^ cells of NL4-3 HIV-1 (NIH AIDS Reagent Program) pseudotyped with vesicular stomatitis virus glycoprotein, washed, and placed back in culture in complete medium without chemokine or cytokines. To activate the virus, latently infected CD4^+^ T cells were exposed to 10 µg/ml PHA, 1 μM PRO, or 10 nM BRY, or not stimulated (ns) at day 3 post-infection. Finally, at 72 h post-stimulation, cells were harvested and analyzed by FACS for intracellular p24 accumulation and cell-surface NKG2DLs expression.

### RT-qPCR

Total RNA was extracted with TRIzol (Life Technologies) from latently infected and control non-infected CD4^+^ T cells 24 h after treatment with 1 μM PRO, 10 nM BRY, or medium alone (ns). Aliquots (2 µg) of total RNA were used to generate cDNA using random hexamers, and the resulting cDNA (25 ng) was amplified in triplicate using the SensiFAST SYBR Green PCR master mix (all from Bioline). The qPCR reactions were performed using primers for MICA, MICB, ULBP2, and PIGS as previously described (32).

### NK-Cell-Mediated Killing of Reactivated HIV-1-Infected Cells

Primary HIV-1-infected CD4^+^ T cells (targets) reactivated with 1 μM PRO or 10 nM BRY or nt were collected 54 h post-stimulation and further cultivated for 18 h in the same treatment conditions (nt, PRO, or BRY) either alone or together with NK cells (effectors) purified from a cryopreserved aliquot of PBMCs of the same donor at an E:T ratio of 1:1. Prior to incubation with targets, NK were treated with saturating amounts of anti-NKG2D blocking antibody (1 µg/10^6^ cells) or control IgG_1_ for 15 min. Cells in the co-culture were then fixed/permeabilized and stained for p24 and CD3. Finally, 7,000 target cells (gated as CD3^+^) were acquired by FACS. The percent NK-cell-mediated killing of p24^+^ cells was calculated with the following formula: 100 × (% p24^+^ cells in targets − % p24^+^ cells in targets with effectors)/(% p24^+^ cells in targets).

### Statistical Analysis

All experiments have been performed independently at least three times. GraphPad Prism 6.0 software was used to perform all statistical analyses. A value of *P* < 0.05 was considered statistically significant.

## Results

### PRO and BRY Activate NK Cells Affecting Their Phenotype

**Figure 1:**
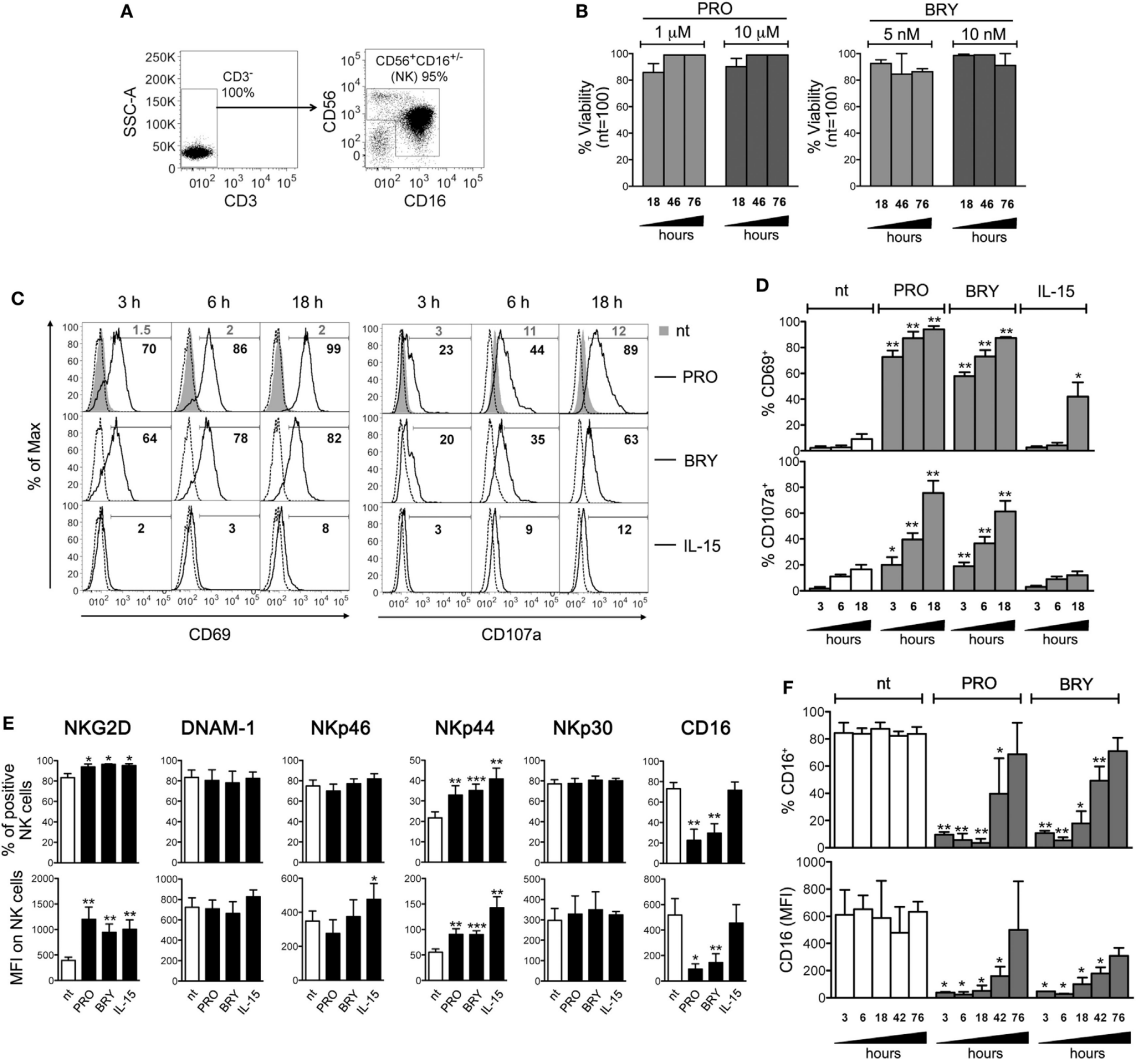
Prostratin (PRO) and bryostatin-1 (BRY) activate natural killer (NK) cells and modulate activating receptors. **(A)** The purity of NK cells isolated from PBMCs was examined by flow cytometry measuring the frequency of CD3^−^CD56^+^CD16^+/−^ cells. **(B)** NK cells were cultured for 18, 46, and 76 h in medium alone [not treated (nt)] or supplemented with 1 or 10 µM PRO, or with 5 or 10 nM BRY. The viability of gated NK cells was examined by LIVE/DEAD staining and expressed relatively to nt cells set at 100%. Bars represent mean ± SEM (*n* = 3). **(C–F)** NK cells treated or not with 1 µM PRO, 10 nM BRY, or 12.5 ng/ml of IL-15 were examined at various time points as indicated **(C,D,F)** or after 18 h only **(E)** for the expression of various markers: **(C,D)** the frequency of CD69^+^ and CD107a^+^ NK cells among nt (filled gray histograms, gray percentages) and treated cells (open histograms, black percentages) is shown together with control IgG signal (dashed line) for a representative experiment **(C)** and as mean ± SEM **(D)**; **(E,F)** mean ± SEM of both percentage of positive cells and mean fluorescence intensity (MFI) for NKG2D, DNAM-1, NKp46, NKp44, NKp30, and CD16 is shown. Experiments were performed with at least three independent donors [up to eight in panel **(E)**]. *P < 0.05, **P < 0.01, and ***P < 0.001 by paired *t* test.

Primary NK cells were isolated from PBMCs of healthy donors by negative selection with an average purity of 92 ± 2% (mean ± SD) calculated as percentage of CD3^−^CD56^+^CD16^+/−^ cells by FACS analysis (Figure 1A), and cultivated for 18, 46, and 76 h in the presence of PRO or BRY at concentrations that effectively reverted HIV-1 latency in *in vitro* models (1 and 10 µM PRO, 5 and 10 nM BRY) (10, 46–48), or without drugs (nt). In all conditions, the viability of NK cells was not significantly affected (Figure 1B), thus we decided to use for most experiments in this study the lowest dose (1 µM) of PRO for which toxicity is not known, and the 10 nM concentration of BRY that is required for *ex vivo* latent HIV-1 reactivation (9, 11). First, we evaluated the cell-surface expression of CD69 and CD107 markers of cell activation and degranulation, respectively, after 3, 6, and 18 h of culture in the presence of PRO, BRY, or a physiologic stimulation, i.e., 12.5 ng/ml of IL-15. As shown in Figures 1C,D, both drugs induced NK cell activation more rapidly (i.e., clearly detectable after 3 h) and to a much higher extent, especially PRO, if compared with IL-15 in terms of frequency of CD69^+^ cells (9 ± 4, 94 ± 3, 85 ± 0.1, and 42 ± 11% after 18 h in nt, PRO, BRY, and IL-15 cultures, respectively; *n* = 4, mean ± SEM). In addition, the basal percentage of CD107a^+^ cells that underwent degranulation in nt cultures did not increase in the presence of IL-15 (17 ± 4 vs 12 ± 4% at 18 h), but was rapidly and potently enhanced by the addition of PRO and BRY, the former having the strongest effect (76 ± 9 and 61 ± 8%, respectively, at 18 h). Moreover, by measuring after 18 h the cell-surface expression of various NK-cell-activating receptors, we found that both PRO and BRY shared the capacity of IL-15 to upmodulate NKG2D and NKp44 in terms of frequency of expressing cells as well as receptor MFI, while none of the treatments changed basal expression of DNAM-1, NKp46, and NKp30 (Figure 1E). On the other hand, exposure to PRO or BRY resulted in a drastic reduction of CD16 (FcγRIIIa) receptor levels both as % CD16^+^ cells (22 ± 11 and 30 ± 9, respectively, vs 73 ± 6% in nt cells; *n* = 8) and CD16 MFI (93 ± 41 and 145 ± 69, respectively, vs 517 ± 129% in nt cells), while IL-15 had no effect (Figure 1E). Downregulation of CD16 by PRO or BRY was also monitored at various time points (from 3 to 76 h of treatment), showing that the effect of the drugs was very rapid, starting at 3 h in parallel with CD69 and CD107 upregulation and reaching a maximum between 6 and 18 h (Figure 1F). At later time points, the cell-surface expression of CD16 was progressively reconstituted and completely resumed at 76 h (Figure 1F). Overall, results indicate that PRO or BRY induce activation of NK cells more potently than IL-15 resulting in rapid degranulation and loss of CD16 expression. In addition, PRO or BRY increased expression of NKG2D and NKp44 activating receptors similar to IL-15.

### PRO and BRY Downregulate CD16 on NK Cells *via* Matrix Metalloprotease (MMP)-Mediated Shedding

Previous studies have shown decreased CD16 expression on NK cells activated with strong stimuli such as PMA, two cytokine combination (e.g., IL-12 plus IL-18), triggering *via* anti-CD16 antibody or co-culture with transformed or antibody-coated cell targets (49–53). Through the use of chemical inhibitors, the activity of MMPs was shown to be crucial for CD16 downregulation in NK cells activated with different stimuli (50–53), while the function of protein kinases including PKC is unclear, as yet, being required for the downregulation of CD16 by PMA but not by anti-CD16 antibody (49).

**Figure 2:**
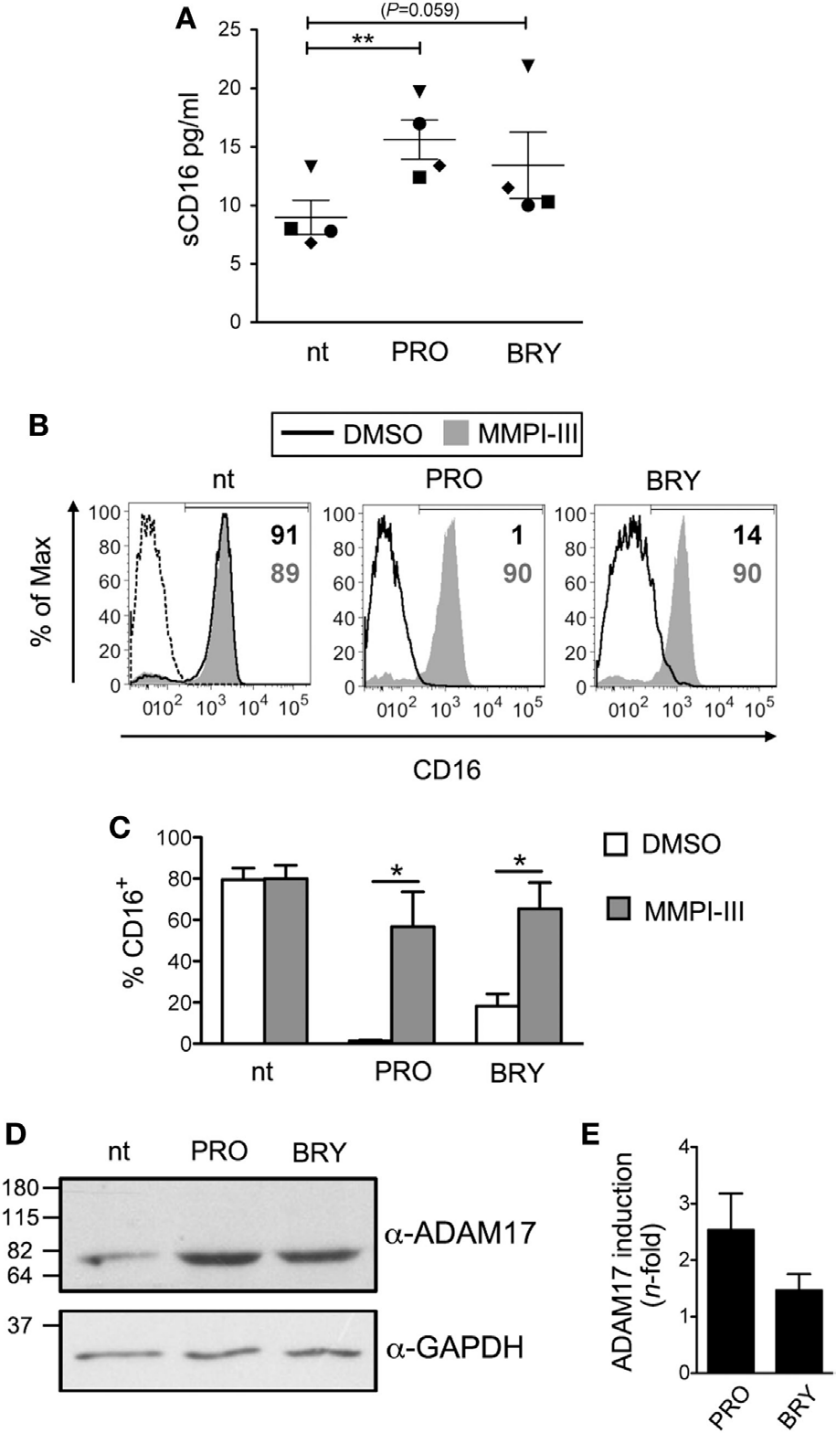
CD16 is shed from natural killer (NK) cells upon exposure to prostratin (PRO) or bryostatin-1 (BRY). **(A)** Soluble CD16 (sCD16) was measured in the media of NK cells after 18 h culture without [not treated (nt)] or with the addition of 1 µM PRO or 10 nM BRY. Each symbol represents one donor (*n* = 4). The mean ± SEM is reported. **(B,C)** NK cells were pre-exposed to 25 µM MMPI-III inhibitor or equivalent amounts of DMSO for 1 h, then 1 µM PRO or 10 nM BRY were added or not for 18 h and finally analyzed for cell-surface CD16 expression. **(B)** Histograms show CD16 signal on nt, PRO- and BRY-treated cells either exposed to MMPI-III (filled gray histograms) or to DMSO (solid line) in one representative experiment. Signal of control IgG (dashed line) and the frequency of CD16^+^ cells (gray or black when MMPI-III or DMSO are present, respectively) are also shown. **(C)** Bars represent mean ± SEM (*n* = 3). **(D)** The catalytic 80 kDa form of ADAM17 (top) and GAPDH as internal control (bottom) were detected by western blotting analysis of total protein extracts of nt, PRO- and BRY-treated NK cells. Molecular mass standards are indicated (kDa). **(E)** Quantification of ADA17 induction by PRO and BRY relative to nt condition. The ADA17 signal normalized for GAPDH signal was quantified and divided for the value obtained in the nt sample. Data show mean ± SEM of three independent experiments like the one shown in **(D)**. *P < 0.05 and **P < 0.01 by paired *t* test.

Then, to investigate whether PRO and BRY induced proteolytic shedding of CD16 from NK cells, we measured by ELISA the amount of soluble CD16 (sCD16) released in the extracellular medium after 5 h of treatment. Results showed that purified NK cells spontaneously release some sCD16 in the culture medium (9 ± 1 pg/ml, *n* = 4; Figure 2A) and that this amounts were consistently higher if PRO was present in the culture (16 ± 2 pg/ml, *P* = 0.007). A trend toward increased sCD16 levels was also observed in cultures of NK cells supplemented with BRY (13 ± 3 pg/ml, *P* = 0.059). Next, to determine whether MMPs play a role in CD16 shedding induced by PRO and BRY, a broad spectrum MMP inhibitor (MMPI-III, 25 µM) or equivalent amounts of solvent (DMSO) were added to NK cells in 18-h cultures together with PRO or BRY or in the absence of these drugs (nt). Both MMPI-III and DMSO had no effect on NK cell viability (data not shown) or on basal CD16 expression on nt cells (Figures 2B,C). On the other hand, the inhibitor, not its solvent, completely abolished CD16 downregulation by PRO or BRY, as indicated by full restoration of CD16^+^ NK-cell percentage (Figures 2B,C). Moreover, given that disintegrin and metalloprotease 17 (ADAM17) is the principal MMP involved in CD16 shedding in NK cells activated with PMA or IL-12/IL-18 (53, 54), we analyzed by western blotting the levels of ADAM17 expression in NK cells treated for 18 h with PRO, BRY, or untreated. Of note, the mature catalytic form (80 kDa) of ADAM17 was detected at higher intensity in extracts of PRO- and BRY-treated cells if compared with nt cells, with a median induction of 2.5- and 1.5-fold, respectively, in three independent experiments (Figures 2D,E). These results suggest that CD16 downregulation induced by PRO and BRY in NK cells results from the activity of cellular MMPs including ADAM17 that mediate CD16 cleavage and release from the cell membrane.

### Impact of PRO and BRY on NK-Cell Functionality

**Figure 3:**
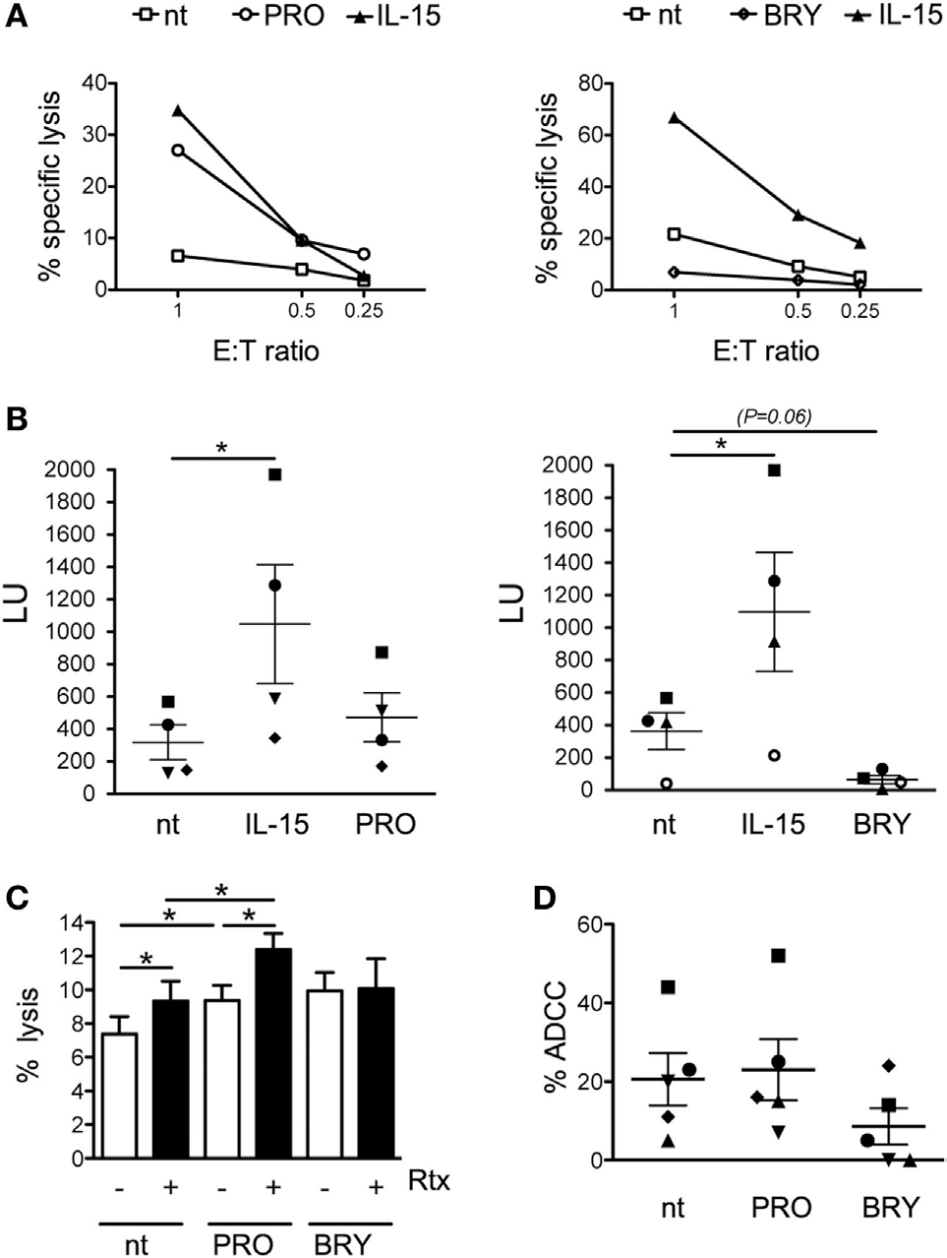
Impact of prostratin (PRO) and bryostatin-1 (BRY) on natural killer (NK)-cell cytotoxicity. **(A)** NK cells cultivated for 18 h without stimuli [not treated (nt)], with 12.5 ng/ml of IL-15, 10 µM PRO or 5 nM BRY were tested for cytotoxicity against K562 cell targets at the indicated E:T ratio. The percent specific lysis of two representative experiments is shown. **(B)** The NK cell-mediated lysis measured in four independent experiments like the one shown in panel **(A)** was converted in lytic units (LU, mean ± SEM). Each symbol represents one donor. **(C,D)** NK cells treated for 18 h with 1 µM PRO or 10 nM BRY or nt were tested against Raji cells coated or not with rituximab (Rtx) monoclonal antibody. The efficiency of overall lysis **(C)** and antibody-dependent cellular cytotoxicity **(D)** were measured in five donors. The mean ± SEM is reported. *P < 0.05 by paired *t* test.

Next, we assessed whether PRO or BRY treatment has an impact in the cytotoxic function of NK cells. First, NK cells purified from four healthy donors were cultivated for 18 h with either PRO or BRY and, in parallel, with IL-15 or without stimuli (nt), then incubated in a 4-h lysis assay at various effector-to-target (E:T) ratio with K562 cells, a tumor cell line that serves as established target of NK cytotoxicity. Pre-treatment with IL-15 consistently boosted NK-cell cytotoxic activity if compared with nt control (Figures 3A,B). A similar effect was observed for PRO in two donors (one is shown in Figure 3A), but the median cytotoxicity of PRO-treated NK cells of four donors did not differ significantly from that of nt cells (Figure 3B). Conversely, BRY did not stimulate NK-cell activity but rather inhibited cytotoxicity in three donors (an example is shown in Figure 3A), resulting in a nearly significant fourfold inhibition across all donors (Figure 3B; *P* = 0.057). Moreover, as CD16 downregulation may affect antibody-mediated recognition of targets, we compared the ADCC activity of nt NK cells with that of PRO- or BRY-treated NK cells. To this end, we set up a FACS-based cytotoxicity assay using PBMCs freshly isolated from intravenous blood samples and cultivated for 18 h with or without drugs as effectors and Raji CD20^+^ Burkitt’s lymphoma cells, pre-coated or not with Rituximab (Rtx, a recombinant anti-CD20-human IgG_1a_), as targets. In pilot tests and throughout ADCC experiments, we found that CD16 downregulation on NK cells by PRO and BRY also occurred when using the whole PBMC population rather than purified cells (data not shown). Results obtained with five donors showed that coating with Rtx increased significantly the overall susceptibility of Raji cells to lysis mediated by NK cells treated with PRO or nt, but not by BRY-treated NK cells (Figure 3C). More specifically, the % ADCC activity of nt NK cells was increased in 4/5 donors and in only 1/5 donors by pre-treatment with PRO and BRY, respectively (Figure 3D). On the other side, PRO and BRY treatment reduced ADCC of NK cells 1/5 and 4/5 donors, respectively (Figure 3D). Therefore, these drugs may either stimulate or inhibit ADCC, likely as a result of the balance between opposite effects (i.e., NK-cell activation and CD16 downregulation; see Discussion). Due to donor variability, the average % ADCC activity of NK cells treated with BRY or PRO did not differ significantly from that of untreated NK cells (Figure 3D).

Overall, despite considerable variability exists among individuals as to NK cell functionality in response to PRO and BRY, our data suggest that both cytotoxicity against tumor targets and ADCC activity of NK cells are generally maintained or even enhanced after exposure to PRO but are frequently impaired upon exposure to BRY.

### PRO Upregulates ULBP2 Expression on CD4^+^ T Cells With Reactivated HIV

**Figure 4:**
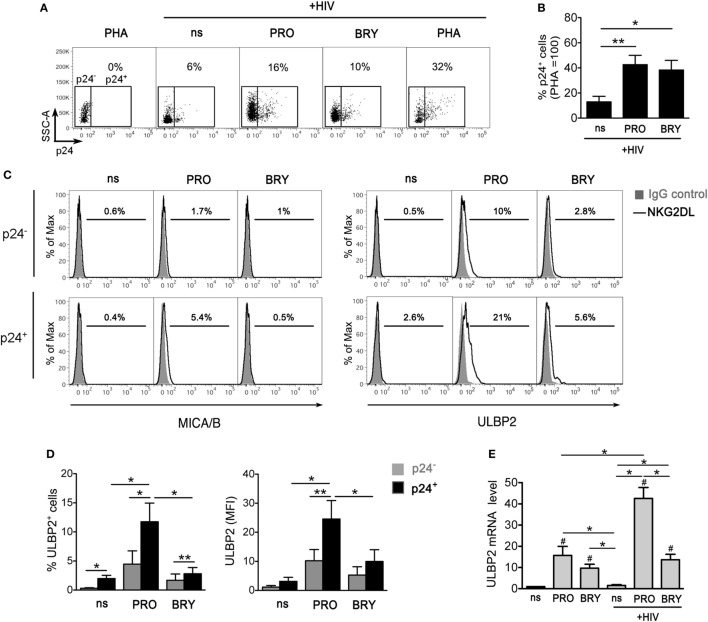
ULBP2 is induced on latently infected CD4^+^ T cells upon HIV-1 reactivation by prostratin (PRO) and, to a lesser extent, by bryostatin-1 (BRY). To establish viral latency, freshly isolated CD4^+^ T cells were cultivated in the presence of CCL19 for 1–3 days, infected or not with HIV-1, and further cultured for 3 days, as described in details in Section “Materials and Methods.” Then, cells were stimulated with 10 µg/ml phytohemagglutinin (PHA), 1 µM PRO, or 10 nM BRY, or not stimulated (ns), further cultivated for 3 days and finally analyzed by two-color flow cytometry for the expression of intracellular p24 and cell-surface MICA/B and ULBP2. **(A)** Representative dot plots show the frequency of reactivated p24^+^ cells gated on non-infected PHA-stimulated control cells. **(B)** Percentage of p24^+^ cells was determined as shown in panel **(A)** in four independent experiments and normalized to HIV-infected PHA-treated cultures (mean ± SEM). **(C)** Histograms show MICA/B and ULBP2 fluorescence in the gated p24^−^ (top panels) and p24^+^ (bottom panels) cell populations measured in a representative experiment on ns, PRO- and BRY-stimulated cell samples. Signals obtained with control IgG (filled histograms) and the percentage of ligand-positive cells are shown. **(D)** ULBP2 expression (mean ± SEM), both% of positive cells and mean fluorescence intensity (MFI), was determined as shown in panel **(C)** in four independent experiments. **(E)** RT-qPCR was used to assess ULBP2 mRNA levels in latently infected and in control non-infected CD4^+^ T cells cultivated for 24 h in ns, PRO, and BRY conditions. Results are expressed relative to ns non-infected sample (set to 1). Mean ± SEM values obtained in three independent experiments performed in duplicate are shown. *P < 0.05 and **P < 0.01 by paired *t* test. Values that differ significantly from ns non-infected samples in panel **(E)** are also indicated (^#^*P* < 0.05 by paired *t* test).

We recently demonstrated that Vorinostat (SAHA) treatment of CD4^+^ T lymphocytes latently infected with HIV-1 induces the expression of NKG2D ligands (NKG2DLs), specifically ULBP2 and, to a lesser degree, of MICA/B, on those cells that become p24^+^ (HIV-1 Gag capsid antigen), hence providing a proof of principle that reactivated HIV-1 upregulates NKG2DLs and exposes infected cells to NKG2D-mediated killing by NK cells (32). To extend this analysis to PKCas, we investigated NKG2DLs expression on CD4^+^ T cells following HIV-1 reactivation by PRO or BRY. Latent HIV-1 infection was established in primary resting CD4^+^ T cells pre-treated with CCL19 according to a previously reported protocol (see Materials and Methods), then cells were exposed for 72 h to 1 µM PRO, 10 mM BRY, or, as controls, activated with 10 µg/ml of PHA or not stimulated (ns), prior analysis of intracellular p24 and cell-surface MICA/B and ULBP2 expression by two-color flow cytometry (representative and summary data with four donors are shown in Figures 4A–D). We found that treatment with PRO and BRY induced the appearance of 19 ± 6 and 15 ± 5% p24^+^ cells, respectively, less efficiently than PHA (30 ± 11%), yet well above the levels due to endogenous viral reactivation in ns cultures (4 ± 2%) (Figures 4A,B). In addition, in line with the capacity of HIV-1 to upregulate NKG2DLs, particularly ULBP2 (27–31), we found that expression of ULBP2 was higher on p24^+^ if compared with p24^−^ cells (Figures 4C,D). On the other hand, MICA/B expression was either absent or slightly increased on p24^+^ cells (Figure 4C shows a positive example), as previously observed in Vorinostat-treated cultures (32), which may be related to the elevated genetic polymorphisms and inter-individual variability of MIC protein levels (55, 56). Interestingly, the upregulation of ULBP2 on p24^+^ vs p24^−^ cells consisted in a modest increase of % ULBP2^+^ cells in nt and in BRY-stimulated cultures, while it was significantly higher in PRO-stimulated cultures both as % ULBP2^+^ cells and ULBP2 MFI on p24^+^ cells (Figure 4D). Therefore, although PRO and BRY display similar latency reversal activity, only PRO can further enhance ULBP2 expression induced by reactivated HIV-1. To gain insight into the mechanism, RT-qPCR analysis of ULBP2 mRNA was performed in CD4^+^ T cells that have been either latently infected with HIV-1 or non-infected, then cultivated for 24 h in medium alone (ns) or supplemented with PRO or BRY. Figure 4E shows that latent HIV-1 infection resulted in a ~1.5-fold increase of ULBP2 mRNA levels in ns CD4^+^ T cells, albeit not statistically significant, whereas the sole exposure to PRO or BRY lead to a strong ULBP2 mRNA upregulation in non-infected cells (by 16- and 10-fold, respectively); moreover, ULBP2 mRNA was further increased (up to 43-folds) when PRO but not BRY was added to latently infected cell cultures. These results suggest that PRO and BRY upregulate ULBP2 at the transcriptional level and that only PRO synergizes with reactivated HIV-1 at inducing ULBP2 expression.

### The Susceptibility to NK-Cell-Mediated Killing of HIV-Infected CD4^+^ T Cells That Exit From Latency Is Increased by PRO

**Figure 5:**
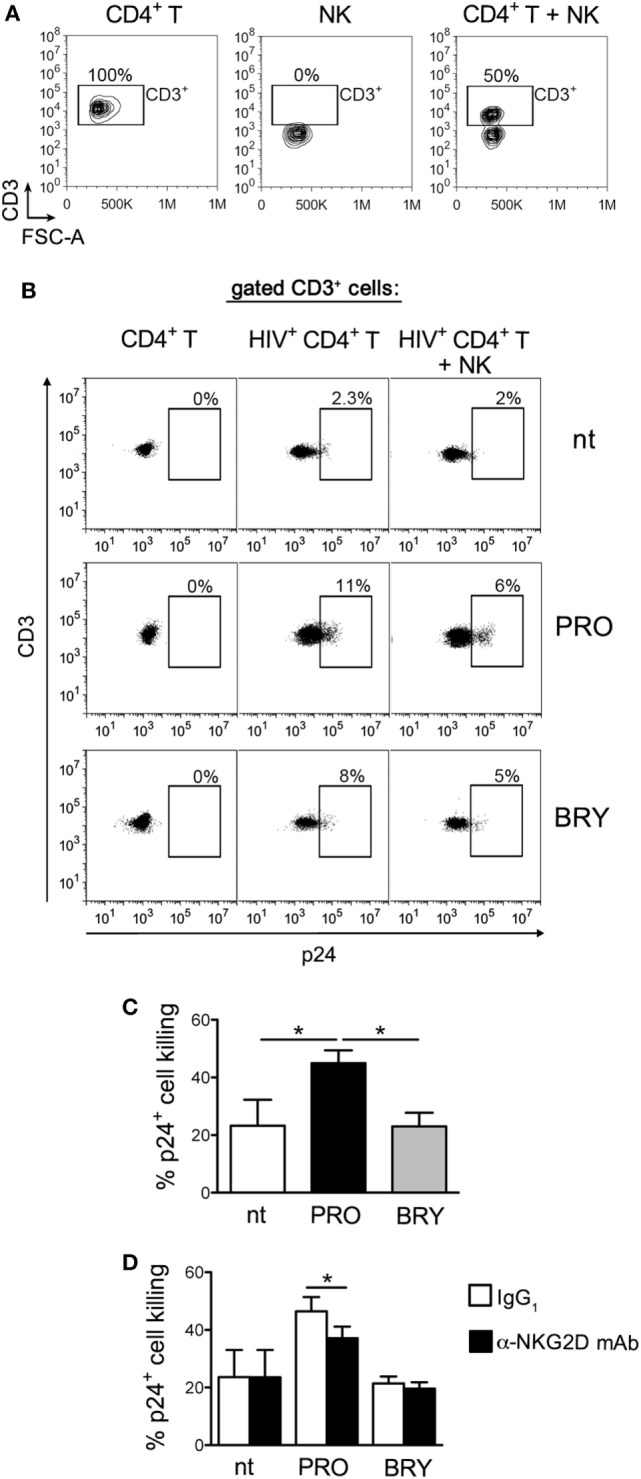
Prostratin (PRO) enhances the capacity of natural killer (NK) cells to suppress HIV-infected CD4^+^ T cells that exit from latency. A co-culture assay of latently infected CD4^+^ T cells at day 2 post-stimulation with 1 µM PRO or 10 nM bryostatin-1 (BRY) and autologous NK cells at a 1:1 E:T ratio was performed overnight (18 h) in the presence of the same drug. Then, cells were analyzed to measure the frequency of p24^+^ cells among gated CD3^+^ targets and calculate % reduction of 24^+^ targets by NK cells. **(A)** The CD3^+^ cell gate was set on cultures of targets alone, so that effectors cells were excluded, as shown in control cultures of effectors alone as well as in co-cultures of targets and effectors in a representative experiment. **(B)** A representative set of results with PRO- and BRY-exposed cultures as well as control not treated (nt) cultures is shown. **(C)** The NK-cell-mediated p24^+^ cell killing was measured in seven independent experiments and expressed as % 24^+^ killing (mean ± SEM). **(D)** NK cells were preincubated with anti-NKG2D monoclonal antibody (mAb) or isotype control IgG_1_ immediately before the overnight co-culture with targets in nt, PRO, and BRY conditions, then the % 24^+^ killing was calculated in three independent experiments. *P < 0.05 by paired *t* test.

Finally, we investigated the overall impact in NK-cell-mediated killing of latently infected CD4^+^ T cells when both effector and target cells are exposed to PRO or BRY. To this end, we set up a p24^+^ cell reduction assay using autologous NK and CD4^+^ T cells purified from healthy donors (see Materials and Methods). In brief, freshly isolated resting CD4^+^ T cells were infected with HIV-1 to establish viral latency, then cells were treated or not with PRO or BRY as described above. After 54 h of treatment, cells were collected, resuspended in the same treatment condition (nt, PRO, or BRY) either alone or together with NK cells purified from a cryopreserved aliquot of PBMCs of the same donor at an E:T ratio of 1:1, and cultivated for further 18 h. Finally, the frequency of p24^+^ cells was measured by flow cytometry within the target cell population gated as CD3^+^ (Figures 5A,B show the gating strategy). By comparing CD4^+^ T with CD4^+^ T + NK cultures for seven donors, we found that the frequency p24^+^ cells was similarly reduced by the presence of NK cells in nt and BRY cultures (23 ± 9 and 23 ± 5% reduction, respectively), while a significantly stronger reduction was observed in cultures of cells exposed to PRO (45 ± 5%) (Figures 5B,C show a representative set of results and summary data, respectively). These results indicate that, when both effectors and targets are treated, PRO but not BRY can enhance the NK-cell-mediated clearance of the HIV-1 reservoir in CD4^+^ T cells. In this assay, the contribution of NKG2DLs to target cell lysis was investigated by preincubating effectors cells with an anti-NKG2D mAb or with isotype control IgG. In line with ULBP2 expression on targets, NK cell-mediated killing of p24^+^ cells was significantly reduced by the anti-NKG2D mAb only in the presence of PRO (Figure 5D).

## Discussion

To select drugs for “shock-and-kill” interventions, it is important to evaluate the efficacy at reversing HIV-1 latency as well as the absence of deleterious effects on immune effector cells such as those recently reported for various LRAs (19, 20, 40, 57). In this study, we investigated two LRAs belonging to the group of PKCa, PRO and BRY, for their impact on NK cell function, as the potential of NK cells in HIV-1 eradication was lately underscored (33–36).

Although the capacity of PRO and BRY to disrupt HIV-1 latency *in vivo* still awaits demonstration, various lines of evidence make these drugs appealing LRA candidates. In fact, studies performed *in vitro* demonstrated that PRO and BRY efficiently reverse HIV-1 latency in model systems based on T cell lines as well as primary CD4^+^ T cells (10, 46–48) and, differently from other LRAs, are also effective at inducing HIV-1 transcription and viral particle release in cells isolated from ART-treated patients (9–11). This is consistent with the fact that stimulation of the PKC pathway induces proviral expression by activation of NF-κB and AP-1 that crucially regulate HIV-1 LTR transcription (6). Specifically, PRO was shown to induce latent HIV-1 expression *via* PKC-mediated phosphorylation and degradation of IκBα, leading to the translocation of NF-κB into the nucleus where it transactivates the viral LTR (47, 58). As for BRY, its activity on latent HIV-1 was associated with activation of the PKC pathway *via* the adenosine monophosphate-activated kinase (59). An additional important feature shared by PRO and BRY is the potential to inhibit *de novo* infection by reactivated virus through the capacity to downregulate the HIV-1 receptor CD4 as well as CCR5 and/or CXCR4 co-receptors (46, 58, 59).

Here, we demonstrated that PRO and BRY are potent activators of NK cells without affecting their viability. When compared with stimulation with IL-15, PRO and BRY upmodulate NKG2D and NKp44 to similar levels, but induced a faster and more efficient degranulation and CD69 upregulation, furthermore lead to rapid loss of CD16 expression analogously to strong stimuli such as PMA or two-cytokine combinations (53, 54). These results are partially consistent with a recent study showing that treatment of NK cells with PRO resulted in higher cellular activation and NKG2D expression, in lower CD16 levels, but not in upregulation of NKp44 (40). Here, we further investigated CD16 modulation and its impact on NK cell function. We showed that CD16 downregulation by PRO or BRY was due to enhanced sCD16 shedding mediated by MMP enzymes which, most likely, included ADAM17. Indeed, the catalytic form of ADAM17, that specifically cleaves CD16 among other substrates (54), accumulated within NK cells after exposure to PRO or BRY, in agreement with a previous report describing increased ADAM17 mRNA levels by a gene array analysis of THP-1 cells treated with PRO (58). Besides CD16, various receptors, cytokines, and adhesion molecules are validated ADAM17 substrates, including molecule with important function in immune cell responses such as TNF-α and CD62L (60), hence upmodulation of ADAM17 induced by PKCa may have a multifaceted effect in the biology of NK cells.

While NK-cell activation and upregulation of NKG2D and NKp44 may result in increased cytotoxicity against tumors or infected cells, CD16 downregulation could hinder ADCC activity of NK cells. However, although the phenotypic changes induced by PRO and BRY were equivalent and reproducibly observed in NK cells of all tested donors, heterogeneous results were obtained by functional assays performed during this study.

First, we found donor-to-donor differences in the impact of PRO and BRY on NK-cell cytotoxicity against K562 cells in four tested donors: in half the subjects PRO-stimulated cytotoxicity but had no effect in the other half, while BRY inhibited cytotoxicity in three subjects and had no effect in one subject. Hence, in those individuals who responded to treatment, the natural cytotoxicity was increased by PRO and, unexpectedly, inhibited by BRY. In addition, the ADCC activity against antibody-coated Raji cells of NK cells from five donors was repeatedly impaired by BRY (in four of five donors) but not by PRO (in one of five donors). Remarkably, when not inhibited, ADCC was rather stimulated (in one of five and four of five donors with BRY and PRO, respectively). These results suggest that NK-cell ADCC can be elicited by very low CD16 amounts and that this activity is positively regulated by PKCas at levels distinct from CD16 cell-surface density. Indeed, the activity of PKC is required for ADCC, as this function is impaired in NK cells pre-treated with a PKC inhibitor (38). Here, we found that, along with CD16 shedding, PRO and BRY stimulate degranulation and induce a generalized activation of NK cells that may potentiate ADCC responses. It is thus possible that the ADCC activity of PKCa-treated NK cells results from the balance between inhibitory (i.e., CD16 downregulation) and stimulatory (i.e., PKC-mediated signaling) events. Apparently, this balance is subjected to inter-individual variation but is more often shifted toward inhibition with BRY and, conversely, activation with PRO.

In addition to investigating the general impact of PRO and BRY on NK cells, we analyzed the effect of these drugs on the capacity of treated NK cells to kill autologous latently infected CD4^+^ T cells reactivated *via* the same treatment. First, we demonstrated that PRO but not BRY enhanced on reactivated p24^+^ cells the upmodulation of ULBP2, a ligand for NKG2D that, when induced by HIV-1 in infected CD4^+^ T cells, activates NK-cell recognition and killing *via* NKG2D triggering (28). These results are consistent with a model that we recently proposed for which the NKG2D/NKG2DLs axis could be exploited to clear HIV-1 reservoirs based on the fact that latent virus and NKG2DLs are under the control of common regulatory pathways and can be simultaneously induced/derepressed by LRAs (32). Here, we showed that both PRO and BRY upregulated ULBP2 at the mRNA level in non-infected CD4^+^ T cells, and that this effect was much higher exclusively for PRO if cells were infected with HIV-1, suggesting that some viral factor(s) synergizes with PRO at stimulating *ULBP2* transcription. Most importantly, we showed that clearance of reactivated p24^+^ cells by NK cells was enhanced when both targets and effectors were exposed to PRO but not to BRY. The stimulatory effect of PRO on NK cell cytotoxicity against PRO-treated p24^+^ targets was repeatedly observed using cells from six donors, thus it was not donor dependent as observed when untreated K562 cells were used as targets (Figure 3B). We hypothesize that ULBP2 upmodulation, invariably induced by PRO in cells harboring reactivated virus, sensitize these cells to recognition and killing by NK cells. In line with this model, the presence of an anti-NKG2D antibody significantly reduced the capacity of NK cells to kill p24^+^ targets in PRO but not in nt or BRY cultures (Figure 5D).

Our results confirmed and expanded data shown in two recent reports that investigated the effects on NK cells of various LRAs including Ingenol-B and PRO, whereas BRY has not been tested. In a study by Garrido and colleagues, pre-treatment of NK cells with PRO resulted in unchanged cytotoxicity against K562 but in increased capacity to suppress HIV-1 replication in a long-term T cell culture, while both activities were impaired by a distinct PKCa, Ingenol-B, or HDACis (40). In another study, latently HIV-1-infected infected CD4^+^ T cells were killed most efficiently in an NK co-culture assay if PRO, not HDACis, was added (57). This latter set of results, however, was undermined by the reported toxicity of PRO on NK cells, even though used at low dose (0.3 µM). The toxic effect of PRO was not seen in our NK co-culture assay, as 1 µM PRO had no effect on the viability of either NK or T cells (Figure 1 and data not shown), neither it was observed in other reported studies (20, 40). In addition, here we measured the killing activity of NK cells against the most relevant targets for an HIV-1 eradication strategy, namely, reactivated p24^+^ cell targets, rather than against the entire latently infected T cell population (57).

An unexpected disclosure of this study consists in the fact that PRO and BRY had opposite effects on various NK cell functions despite these drugs shared similar capacity to activate NK cells or affect their phenotype. Furthermore, PRO and BRY similarly reactivated HIV-1 in latently infected CD4^+^ T cells, but had diversified effects on the virally induced expression of ULBP2 that was further enhanced exclusively by PRO, with important functional consequences for NK-cell recognition. One possibility is that PRO and BRY have diverse effects on specific PKC isoforms that include conventional (α, β, γ), novel (δ, ε, η, θ), and atypical (ζ, ι/λ) isoforms. To the best of our knowledge, a systematic confrontation of PRO and BRY for their impact on the various PKC isoforms was not performed. One study showed that PKCα and, subsequently, PKCθ are activated by PRO during reactivation of latent HIV-1 (48), conforming to a model in which PKCα acts upstream of PKCθ in the T cell receptor/CD28 pathways (61). Since PKCθ is a key molecule of NK cell intracellular signaling and antitumor immune response (39, 62), its activation by PRO may also account for the stimulatory effect of this drug on NK cell cytotoxicity. In another study, BRY was shown to reactivate latent HIV-1 *via* activation of PKCα and PKCδ, while the specific effect on PKCθ was not investigated (59). Of note, following activation by BRY, membrane-associated PKCα is quickly degraded (within 1 h), more rapidly than with PMA or other PKC activators, and the PKCα protein levels are drastically reduced for at least 72 h, hence antagonizing phorbol esters re-stimulation (59, 63, 64). Therefore, it is conceivable that, following PKCα depletion induced by BRY, NK cells enter in a refractory phase for PKC-regulated signaling pathways that mediate cytotoxicity or ADCC. Further work is required to test whether the diverse effects of PRO and BRY are due to differences in the nature of targeted PKC isoforms and/or on the modality of activating signals.

In summary, here we described distinct effects of BRY and PRO on the function of NK cells that have implications for the inclusion of these drugs in “shock-and-kill” strategies. We have shown that BRY but not PRO inhibited the tumor cell lysis and ADCC activities of NK cells in most tested donors. In addition, by treating both effectors and targets, we showed that PRO enhanced NK cell-mediated killing of T cells harboring reactivated HIV-1, while BRY was devoid of this capacity. Overall, notwithstanding similar LRA activity, in this study PRO outmatched BRY as to the impact on important NK cell functions and on NK-cell-mediated clearance of the HIV-1 reservoir. Of note, analogous results were reported for CD8^+^ T cells in a previous study showing that BRY but not PRO had inhibitory effects on the suppressive capacity of HIV-1-specific CD8^+^ T cells derived from patients (20).

Our study was performed with cells purified from healthy individuals and exposed to PRO and BRY *in vitro*, thus it has limitations. Actually, chronic HIV-1 infection leads to pathologic changes in NK cells, including redistribution of maturation subsets, altered expression of inhibitory and activating receptors, and defective functionality (65). ART-induced suppression of HIV-1 viremia has been reported to normalize subset distribution and phenotype of NK cells, although there is no consensus on the degree of NK-cell function restoration (65). Specifically, some studies described lower NK-cell cytotoxicity (66, 67) or IFN-γ production, especially in ART patients with incomplete recovery of CD4 counts (68, 69). For these reasons, the phenotypic and functional outcomes of PRO and BRY should be confirmed with cells from patients on ART and, ultimately, using *ex vivo* samples collected from PRO or BRY trials.

In order to achieve significant clearance of the HIV-1 reservoir, 2-LRA combinations are under investigation, including combinations of PRO or BRY with a functionally distinct LRA (e.g., HDACi or P-TEFb activator) that result in synergistic activation of latent HIV-1 (10, 11, 70). Various HDACis were shown to inhibit the function of NK and T cells (20, 40, 57), including Romidepsin that showed an adverse effect on T cell activity overriding the stimulatory effect of PRO (20), thus the overall impact of two-LRA combinations on antiviral immune responses should be systematically tested. Moreover, our results emphasize the importance of testing the impact of LRAs on the ADCC activity of NK cells, which was done here for the first time. Indeed, experiments performed *in vitro* (71) or in animal models (72, 73) and indirect evidence in humans (74) indicate that both neutralizing and non-neutralizing anti-HIV-1 antibodies exert destruction of reactivated infected cells *via* ADCC. Therefore, avoiding the co-administration of LRAs that negatively impact ADCC activity by NK cells might be essential for successful application of antibodies or vaccination in the context of “shock-and-kill” strategies. On the other hand, combining LRAs with immunotherapies that contrast inhibitory effects or, as shown for the ALT-803 superagonist of IL-15 (75), potentiate the antiviral function of NK cells, may offer a novel therapeutic opportunity toward HIV-1 eradication. Finally, results presented herein and in a previous report showed considerable donor-to-donor variation of NK cell function in response to treatment with PKCa (this study) or HDACi (57). This variability comes in addition to that observed for the level of LRA-induced HIV-1 reactivation that differed widely between patients on ART, possibly due to viral factors, host factors, or combination of both (12, 76). Hence, future investigation of factors determining patient-to-patient differences in the response to “shock-and-kill” strategies is clearly required.

## Ethics Statement

This study was carried out in accordance with the recommendations of the Policlinico Tor Vergata Ethical Committee with written informed consent from all subjects. All subjects gave written informed consent in accordance with the Declaration of Helsinki.

## Author Contributions

MD conceived the study and wrote the manuscript. MGD, EG, and MD performed experiments and analyzed the data. AF and GA collected blood samples from healthy volunteers after obtaining from them written informed consent. All the authors reviewed the manuscript for content, provided suggestions, and approved the final manuscript.

## Conflict of Interest Statement

The authors declare that the research was conducted in the absence of any commercial of financial relationship that could be construed as a potential conflict of interest.
